# Fisetin and luteolin protect human retinal pigment epithelial cells from oxidative stress-induced cell death and regulate inflammation

**DOI:** 10.1038/srep17645

**Published:** 2015-12-01

**Authors:** Maria Hytti, Niina Piippo, Eveliina Korhonen, Paavo Honkakoski, Kai Kaarniranta, Anu Kauppinen

**Affiliations:** 1 School of Pharmacy, Faculty of Health Sciences, University of Eastern Finland, P.O.B. 1627, FI-70211, Kuopio, Finland; 2 Department of Ophthalmology, Institute of Clinical Medicine, University of Eastern Finland, P.O.B. 1627, FI-70211 Kuopio, Finland; 3 Department of Ophthalmology, Kuopio University Hospital, P.O.B. 100, FI-70029 KYS, Finland

## Abstract

Degeneration of retinal pigment epithelial (RPE) cells is a clinical hallmark of age-related macular degeneration (AMD), the leading cause of blindness among aged people in the Western world. Both inflammation and oxidative stress are known to play vital roles in the development of this disease. Here, we assess the ability of fisetin and luteolin, to protect ARPE-19 cells from oxidative stress-induced cell death and to decrease intracellular inflammation. We also compare the growth and reactivity of human ARPE-19 cells in serum-free and serum-containing conditions. The absence of serum in the culture medium did not prevent ARPE-19 cells from reaching full confluency but caused an increased sensitivity to oxidative stress-induced cell death. Both fisetin and luteolin protected ARPE-19 cells from oxidative stress-induced cell death. They also significantly decreased the release of pro-inflammatory cytokines into the culture medium. The decrease in inflammation was associated with reduced activation of MAPKs and CREB, but was not linked to NF- κB or SIRT1. The ability of fisetin and luteolin to protect and repair stressed RPE cells even after the oxidative insult make them attractive in the search for treatments for AMD.

A state of chronic, subacute inflammation exists in many age-related diseases, such as dementia, arthritis, vascular diseases, and age-related macular degeneration (AMD)^1^. In the elderly, the combination of increased production of reactive oxygen species (ROS) and decreased antioxidant functions, accompanied by an upregulation of several inflammatory genes, such as those coding for interleukin (IL) 1β, IL-6, IL-8, and tumor necrosis factor (TNF) α, leads to a two-pronged attack from altered redox status and dysregulated immune responses^1^,^2^.

Under physiological conditions, the intracellular redox balance and inflammatory reactions are subjected to stringent control by many transcription factors like nuclear factor kappa-light-chain-enhancer of activated B cells (NF-κB) and cAMP-response element binding protein (CREB), as well as signaling proteins of the mitogen-activated protein kinase (MAPK) pathway, such as p38 MAPK, c-Jun N-terminal kinase (JNK), and extracellular signal-regulated kinases (ERK) 1/2^1^,^3^,^4^,^5^,^6^. It is known that increased and/or unbalanced levels of oxidative stress can activate these regulators and evoke a prolonged and unregulated release of inflammatory cytokines^1^,^3^,^7^,^8^,^9^,^10^. This effect is further exacerbated by the fact that pro-inflammatory cytokines, such as IL-6 and TNF-α, are able to activate NF-κB, p38 MAPK, and JNK via an auto-activating loop^1^,^11^,^12^.

The retinal pigment epithelium (RPE) is a single cell layer in the posterior pole of the eye that is involved in maintaining the viability and function of photoreceptors. Due to their high lipid content, active metabolism, and the phagocytosis of photoreceptor outer segments (POS) that are composed of readily-oxidized poly-unsaturated fatty acids, RPE cells are naturally predisposed to oxidative stress generated by lipid peroxidation^10^,^13^. 4-Hydroxynonenal (HNE) is a reactive aldehyde produced during lipid peroxidation and it is known to be the major oxidant present in the retina^14^. The regulation and maintenance of the redox potential by RPE cells are of vital importance because the high oxidative stress burden in the RPE contributes to the activation of MAPKs and NF-κB and subsequently to the increased release of pro-inflammatory cytokines^10^,^15^,^16^,^17^. The combined effect of the prolonged presence of pro-inflammatory cytokines and the direct damage caused by ROS to protein and DNA structures contributes to the degeneration of RPE cells, which marks the onset of the inflammation-associated progressive disease known as AMD.

Fisetin and luteolin, two natural polyphenols found in different plants, including fruits and vegetables^3^,^12^,^18^,^19^,^20^, have been claimed to be able to combat both oxidative stress and inflammation in aged cells. Both compounds exhibit potent anti-oxidative and anti-inflammatory properties^3^,^21^,^22^,^23^,^24^,^25^,^26^. Analyses of the pathways regulated by these polyphenols have shown that fisetin is able to inhibit the activation of NF-κB^18^,^21^,^27^,^28^, ERK1/2^18^,^28^,^29^, p38 MAPK^20^,^21^,^28^, and JNK^20^,^22^,^27^,^28^, as well as the secretion of pro-inflammatory cytokines IL-6 and TNF-α^27^ in a wide variety of different cell types. Similarly, luteolin is an efficient inhibitor of NF-κB^23^,^25^,^30^, ERK1/2^3^,^24^,^26^,^31^,^32^, p38 MAPK^3^,^12^,^26^,^32^,^33^,^34^,^35^, JNK^19^,^26^,^31^,^36^,^37^, as well as being able to block the release of IL-6^31^,^38^ and IL-8^31^. However, the diversity of cell types and the oxidative stimulants used in these experiments seem to exert an influence on the reported efficacy and modes of action of fisetin and luteolin.

The current study has assessed the effects of fisetin or luteolin on RPE cells under high oxidative stress due to an exposure to the lipid peroxidation end-product HNE during stress caused by serum starvation. We have also analyzed the pathways underlying the inflammatory responses in these cells. In order to highlight their therapeutic properties and in contrast to many other studies, polyphenols were added to cell cultures after the exposure to HNE.

## Materials and Methods

### Cell Culture

The human retinal pigment epithelial cell line ARPE-19 was obtained from the American Type Culture Collection (ATCC) and used in all experiments. Cells were routinely cultured in a 1:1 mixture of Dulbecco’s modified Eagle’s medium (DMEM) and nutrient mixture F-12 (Life Technologies, Carlsbad, CA, USA) supplemented with 10% HyClone fetal bovine serum (FBS; Thermo Fisher Scientific, Waltham, MA, USA), 100 U/ml penicillin, 100 μg/ml streptomycin, and 2 mM l-glutamine (Lonza, Basel, Switzerland). Cells were kept in an incubator at +37 °C and 5% CO_2_ in a humidified atmosphere, and passaged with 0.25% Trypsin-EDTA (Life Technologies, Carlsbad, CA, USA) every 3–4 days.

### Cell Treatments

Cell growth experiments were performed by plating ARPE-19 cells on 12-well plates (Sigma-Aldrich, St. Louis, MO, USA) at a concentration of 200,000 cells/ml/well in either serum-free or serum-containing medium. Cells were collected after 6, 12, 24, 48, and 72 h, and counted on a Bürker counter to assess cell numbers. At the same time-points, pictures were taken of the cell cultures with an inverted phase-contrast microscope (Zeiss, Oberkochen, Germany) at a 20× magnification in brightfield. Pictures were also taken once every 10 min between hours 2 and 13 after cell splitting and these images were combined to create a time-lapse movie of cell attachment. In the stimulation experiments, cells were plated on 12-well plates in serum-free medium and incubated for 72 h in a humidified 5% CO_2_ atmosphere at 37 °C before treatments. Alternatively, for studies in fully-differentiated ARPE-19 cells, cells were cultured on mouse laminin (BD, Franklin Lakes, NJ, USA)-coated transwell filters (Sigma-Aldrich, St. Louis, MO, USA) to which they were seeded at a density of 1.6 × 10^5^ cells per cm^2^. These cells were kept for 4 weeks in DMEM:F-12 (1:1) growth medium containing 1% fetal bovine serum. The medium was changed every three to four days. After four weeks, the culture medium was replaced with the serum-free growth medium and cells were serum-starved for 72 hours before treatment. After a wash with fresh serum-free medium, cells were treated with HNE (30 μM, Calbiochem, San Diego, CA, USA) or LPS (10 μg/ml, Sigma-Aldrich, St. Louis, MO, USA) as indicated. After an additional incubation for 1 h, 50 μM of either fisetin or luteolin (both Sigma-Aldrich, St. Louis, MO, USA) was added to the wells. The conditions selected for the stimulations were based on our previous and preliminary studies^39^,^40^. Experiments were performed also with specific inhibitors against p38 MAPK (50 μM, SB203580), MEK1/2 (50 μM, PD98059), or JNK (10 μM, SP600125, all three from Cell Signaling Technologies, Beverly, MA, USA) under the same conditions. Concentrations of MAPK inhibitors were based on preliminary dose finding studies (Suppl. Fig. 1). Untreated cells or cells treated with dimethyl sulfoxide (DMSO, Sigma-Aldrich, St. Louis, MO, USA) at a concentration identical to that present in the fisetin or luteolin samples served as negative controls (0.25% v/v). Cells were incubated for 24 h before medium samples were collected for cytokine measurements and for 4 h or 24 h in case of intracellular protein analyses. Proteins were extracted from cells after a single wash with phosphate-buffered saline (PBS) and subsequent lysis in either the CST-lysis buffer (Cell Signaling Technologies, Beverly, MA, USA) or in the mammalian protein extraction reagent M-PER (Thermo Fisher Scientific, Waltham, MA, USA). All experiments, with the exception of the study in fully-differentiated ARPE-19 cells, were repeated at least three times.

### Small interfering RNA (siRNA) transfections

In the siRNA experiments, cells were plated on 12-well plates in serum-containing antibiotic-free medium at a density of 100,000 cells/ml/well. Cells were incubated for 24 h at +37 °C and 5% CO_2_ in a humidified atmosphere, before being washed once with the siRNA transfection medium (Santa Cruz Biotechnology, Dallas, TX, USA). Specific SIRT1 siRNA or the control siRNA A (both Santa Cruz Biotechnology, Dallas, TX, USA) were transfected into the cells according to the manufacturer’s instructions. Five hours after the transfection, the culture medium was changed to serum-free growth medium. Treatments were performed and samples collected as indicated above at 48 h after the siRNA transfection. Additionally, cell lysate samples were collected in M-PER at 48 h after the transfection to measure the effectiveness of SIRT1 knockdown.

### Cell viability assays

The cytotoxicity of the treatments was measured by determining the activity of lactate dehydrogenase (LDH) enzyme released into medium with the CytoTox96^®^ Non-Radioactive Cytotoxicity Assay (Promega, Fitchburg, WI, USA). The assay was performed according to the manufacturer’s instructions. Additionally, we performed the 3-(4,5-dimethyldiazol-2-yl)-2,5-diphenyltetrazolium bromide (MTT) assay to determine the cells’ metabolic activity, as described previously^41^.

### Enzyme-linked immunosorbent assay (ELISA)

The levels of secreted IL-6 and IL-8 were measured from medium samples with specific BD OptEIA^TM^ human ELISA kits (BD, Franklin Lakes, NJ, USA), and the MCP-1 levels were determined using the human CCL2 (MCP-1) Ready-Set-Go!^®^ ELISA Kit (eBioscience, San Diego, CA, USA). The amounts of phosphorylated p38 MAPK, ERK1/2, JNK1, MEK1, and CREB were measured from cell lysates using specific human PathScan® ELISA Kits obtained from Cell Signaling Technologies (Beverly, MA, USA). DNA binding activity of the NF-κB subunit p65 was measured from cell lysates using a TransAM® Transcription Factor ELISA Kit (Active Motif, Carlsbad, CA, USA). SIRT1 protein levels were measured from cell lysates using the Sirtuin 1 (human) (IntraCellular) ELISA Kit (Adipogen, San Diego, CA, USA). All ELISAs were performed according to the manufacturers’ instructions.

### Western blotting

The protein concentration of the samples was measured using the Bradford protein assay. All samples were normalized to the same protein amount, diluted in a 4× protein loading solution, and separated on a 10% gel for sodium-dodecyl sulfate polyacrylamide gel electrophoresis (SDS-PAGE). Proteins were transferred by electroblotting onto nitrocellulose membranes (Amersham, Piscatawy, NJ, USA) using the wet transfer method. The membranes were blocked in a 5% non-fat milk solution for 1 h at room temperature before being probed with primary antibodies overnight at +4 °C. Primary antibody dilutions were 1:3000 for SIRT1 (Merck Millipore, Billerica, MA, USA), and 1:20000 for GAPDH (Abcam, Cambridge, UK), which was used as an internal loading control. Horseradish peroxidase-linked anti-rabbit secondary antibody (1:10000) was used to detect the primary antibody of SIRT1, while an anti-mouse secondary antibody (1:25000), was used to detect the GAPDH primary antibody. Protein signals were visualized using the Immobilon™ Western Chemiluminescent HRP Substrate (ECL) (Merck Millipore, Billerica, MA) and detected on the Super Rx medical X-ray film (Fuji Corporation, Tokyo, Japan). Band intensity was measured and analyzed using the ImageJ software (U. S. National Institutes of Health, Bethesda, MD, USA; http://imagej.nih.gov/ij/).

### Statistical analysis

Pairwise statistical analyses were performed using the Mann-Whitney U-test in the GraphPad Prism^®^ program (GraphPad Software Inc., San Diego, CA, USA). Results were considered statistically significant at P < 0.05.

## Results

### ARPE-19 cells grow to confluency in serum-free medium

**Figure 1:**
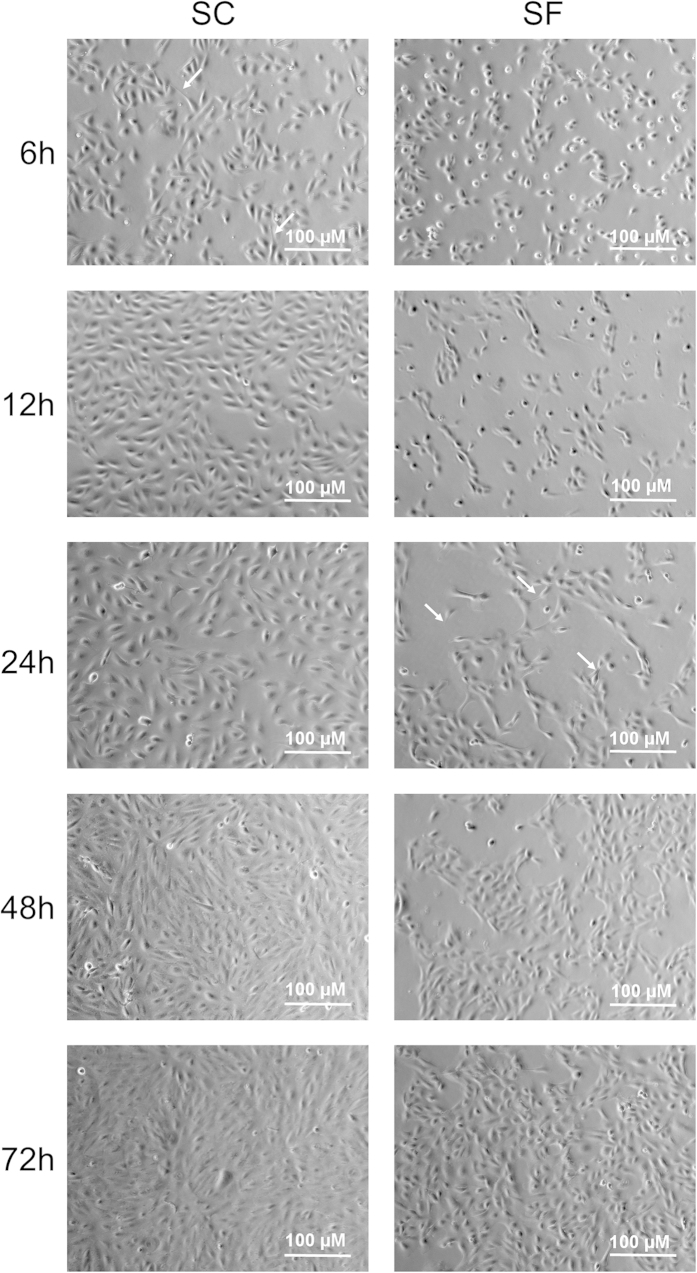
The growth of ARPE-19 cells in serum-containing (SC) or serum-free medium (SF). Cells in serum-free medium were slower to spread out on the surface and start the growth phase but reached confluency after 72 h. White arrows indicate spindle-like protrusions from growing ARPE-19 cells.

In order to induce increased stress, ARPE-19 cells were placed directly after splitting into serum-free medium for all experiments and incubated for up to 72 h in a humidified cell incubator at +37 °C. During the incubation, we monitored the cell growth rate in serum-free medium and compared it to the growth rate of cells maintained under the same conditions in serum-containing medium. Pictures taken 6, 12, 24, 48, and 72 h after plating highlight that it took longer for ARPE-19 cells to adhere to the plate and spread out when cultivated in the serum-free medium when compared to those cultured in the serum-containing medium (Fig. 1). The same phenomenon can also be observed in a time-lapse video of cell attachment (Supplementary material). Those cells cultured in serum-containing medium attached to the surface and spread quicker (Suppl. Video 1) when compared to cells cultured in serum-free medium (Suppl. Video 2). Once they had spread out, however, cells in the serum-free medium grew at a rate similar to that observed for cells cultured in the serum-containing medium (Suppl. Fig. 2). Cells cultured in either medium showed a transient phenotype with spindle-like appearances, which occurred earlier in the cells cultured in serum-containing medium (Fig. 1, white arrows). Irrespective of the presence or absence of serum in the culture medium, ARPE-19 cells reached full confluency and expressed a normal phenotype once confluent.

### Confluent ARPE-19 cells are more susceptible to oxidative stress-related cell death when cultured in serum-free medium

**Figure 2:**
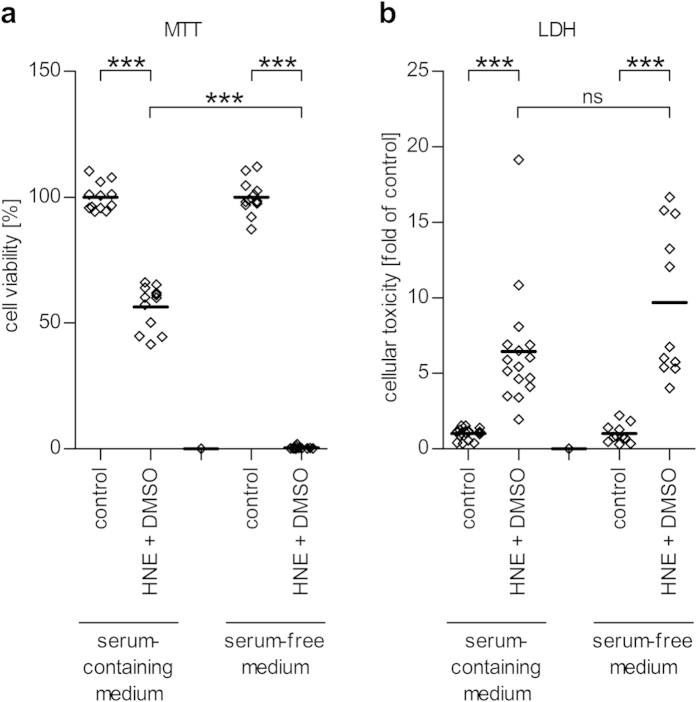
Cytotoxicity of HNE in ARPE-19 cells grown with or without serum measured with the MTT assay (a) and the LDH assay (b). Results are expressed relative to control values of untreated control cells grown in the same medium. Data is combined from three independent experiments with 3–6 replicate samples per group in each experiment, and are represented as scatterplots with median. ns denotes not statistically significant, ***denotes p < 0.001, Mann–Whitney *U*-test.

We examined the differences in the reactions of serum-starved and normally cultured ARPE-19 cells (i.e. in 10% FBS) under increased oxidative stress by culturing both groups of cells in the presence of 30 μM HNE. Our results show that ARPE-19 cells were more susceptible to cell death if they had been serum-starved for an extended period of time (Fig. 2). In the MTT test, exposure of ARPE-19 cells grown in the serum-containing medium to HNE had their cellular viability reduced to 56.4%, whereas cell viability was decreased to only 0.1% in cells grown in the serum-free medium (P < 0.001). In the LDH assay, we observed a substantial release of LDH into the medium after treatment of cells with HNE in both serum-containing and serum-free medium. This indicates that cells cultured in the presence of 10% FBS and treated with HNE retain their metabolic activity better but still suffer from at least transient loss of cell membrane integrity.

### Fisetin and luteolin are able to protect serum-starved ARPE-19 cells from oxidative stress-induced death

**Figure 3:**
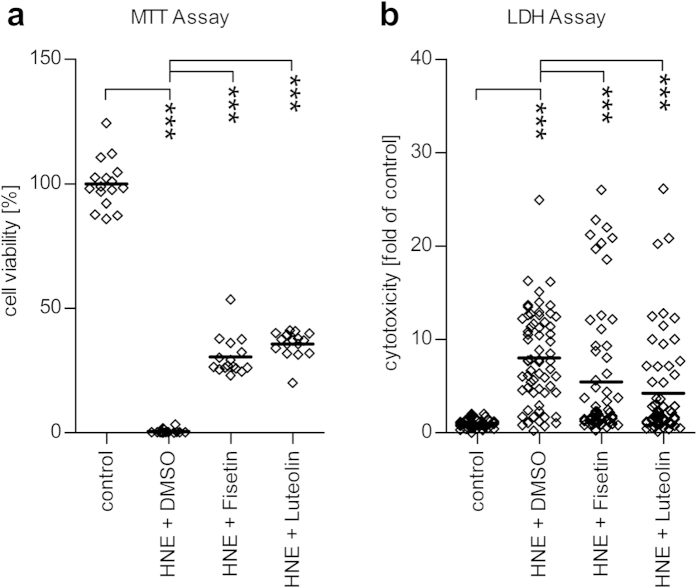
Effect of fisetin and luteolin on the HNE-induced cytotoxicity in serum-starved ARPE cells. Cytotoxicity was determined with the MTT assay (**a**) and the LDH assay (**b**). Both fisetin and luteolin (at 50 μM) improved cell survival after an exposure to HNE and the solvent DMSO. Results are shown as scatterplots with median and are combined from 4–16 independent experiments with 3–4 parallel determinations per group/experiment. ***denotes p < 0.001, Mann–Whitney *U*-test.

We next evaluated whether treatment with either 50 μM fisetin or luteolin could protect ARPE-19 cells from HNE-induced toxicity. Additionally, in order to assess the therapeutic potential of the polyphenols, both fisetin and luteolin were added 1 h after the initial exposure to HNE. Our data show that the polyphenols were able to rescue a significant fraction of serum-starved cells, as measured by the MTT assay (Fig. 3a, p < 0.0001 for both fisetin and luteolin). When compared to untreated controls, the average cell survival rate increased from 0.5% of HNE-treated cells to 30.4% and 35.7% in cells co-treated with fisetin or luteolin, respectively. Similarly to the results in the MTT assay, we found that the release of LDH was significantly lower in cells treated with fisetin or luteolin 1 h after HNE treatment (Fig. 3b, p < 0.0001 for both fisetin and luteolin).

### Fisetin and luteolin inhibit the inflammatory response in stressed ARPE-19 cells

**Figure 4:**
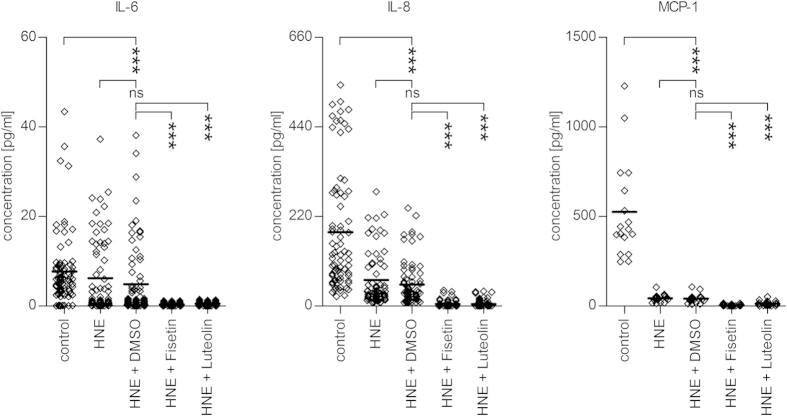
Effect of fisetin and luteolin on the release of cytokines from HNE-treated serum-starved ARPE-19 cells. Both fisetin and luteolin decreased the release of the cytokines IL-6 (**a**), IL-8 (**b**), and MCP-1 (**c**) from ARPE-19 cells when compared to cells exposed to HNE and the solvent DMSO. Results are shown as scatterplots with median and are combined from 5–20 independent experiments with 3–4 parallel determinations per group/experiment. ***denotes p < 0.001, Mann–Whitney *U*-test.

In addition to reduced cellular viability, the exposure of serum-starved ARPE-19 cells to 30 μM HNE decreased the levels of IL-6, IL-8, and MCP-1 when compared to unexposed control cells (Fig. 4). Still, both fisetin and luteolin further decreased the release of IL-6, IL-8, and MCP-1 from HNE-exposed ARPE-19 cells (Fig. 4). The response was highly statistically significant (p < 0.0001 for both fisetin and luteolin in each of the cytokines studied) and lowered the overall levels of released pro-inflammatory cytokines to close to the detection limit. The reduction in IL-6 and IL-8 levels was also observed in fully differentiated ARPE-19 cells cultured for 4 weeks on laminin-coated filter-wells, suggesting that fisetin and luteolin are effective anti-inflammatory agents in both undifferentiated and fully differentiated cells. (Suppl. Fig. 3). The effect of HNE treatment on IL-6, IL-8 or MCP-1 levels was not affected by the polyphenol solvent DMSO (Fig. 4). Therefore, HNE + DMSO-treated cells were chosen to serve as controls to the effects of fisetin and luteolin in this and all subsequent experiments.

### Fisetin and luteolin regulate the phosphorylation of CREB and MAP kinases but do not affect the activity of NF-κB in HNE-exposed ARPE-19 cells

**Figure 5:**
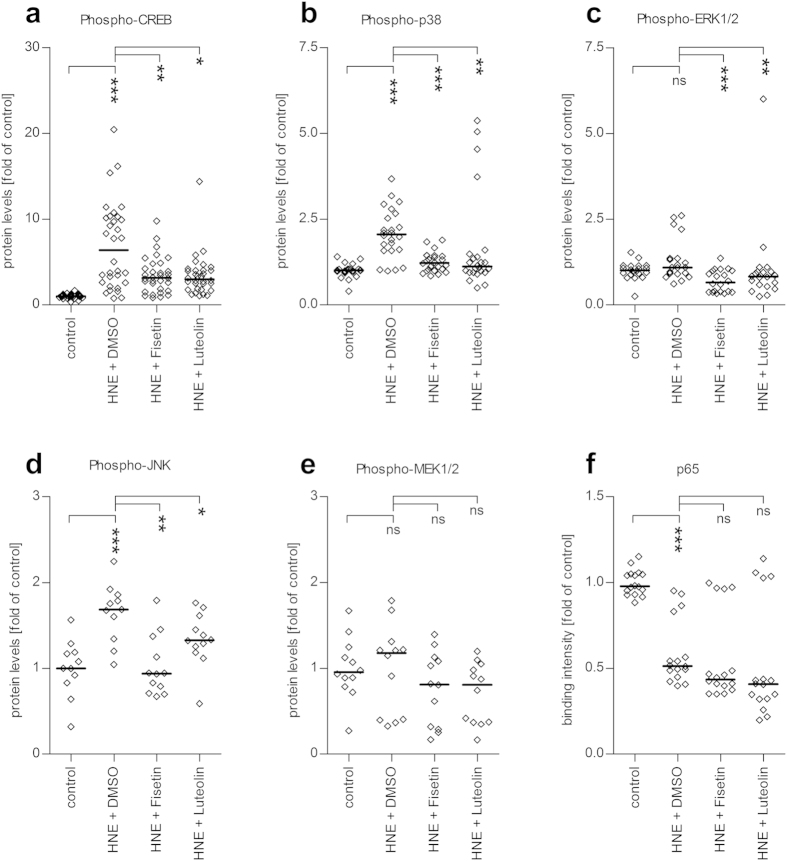
Effect of fisetin or luteolin on signaling pathway proteins. Both fisetin and luteolin reduced the phosphorylation of CREB (**a**), p38 MAPK (**b**), ERK1/2 (**c**), and JNK (**d**), but not that of MEK1/2 (**e**) or the DNA-binding activity of NF-κB subunit p65 (**f**) when compared to cells treated with HNE and DMSO. Results are shown as scatterplots with median and are combined from 3-8 independent experiments with 4 parallel determinations per group/experiment. *denotes p < 0.05, **denotes p < 0.01, ***denotes p < 0.001, ns denotes not statistically significant, Mann–Whitney *U*-test.

Next, we determined the pathways by which fisetin and luteolin were interfering with the production of pro-inflammatory cytokines by measuring the effects of these compounds on the activity of MAP kinases (p38 MAPK, ERK1/2, JNK), and the transcription factors CREB and NF-κB in HNE-treated ARPE-19 cells. Our results show that 4 hours after the fisetin or luteolin treatment, the phosphorylation levels of transcription factor CREB and the MAPKs p38 MAPK, JNK, and ERK1/2 were markedly decreased when compared to cells treated only with HNE and the solvent DMSO (Fig. 5a–d). HNE + DMSO treatment alone increased the levels of phosphorylated CREB, p38 MAPK, and JNK (Fig. 5a,b,d). The phosphorylation level of MEK1/2, the MAP kinase kinase responsible for the ERK1/2 activation, was not statistically significantly affected by HNE exposure, though a slight, statistically non-significant decrease was observed after the treatment with the polyphenols (Fig. 5e, p = 0.141 for fisetin, p = 0.0885 for luteolin). HNE also decreased the binding activity of the NF-κB subunit p65 to its target DNA sequence. Fisetin and luteolin treatments, however, had no additional effect on the DNA-binding activity of p65, suggesting that inhibition of NF-κB is not the main pathway mediating the decrease in the levels of pro-inflammatory cytokines in ARPE-19 cells treated with fisetin or luteolin (Fig. 5f).

### MAPK inhibitors decrease the release of inflammatory cytokines from ARPE-19 cells but do not protect cells from oxidative stress-related toxicity

**Figure 6:**
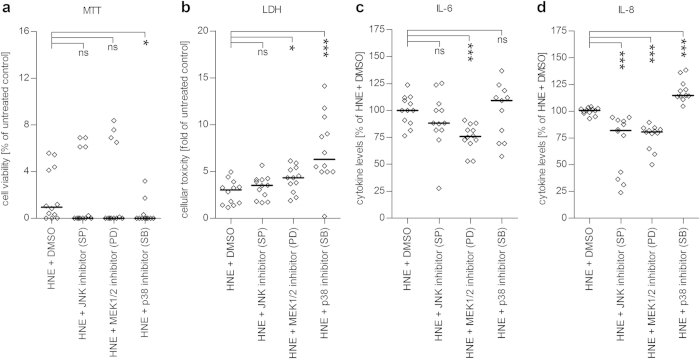
Effects of specific MAPK inhibitors on cell survival and cytokine release from HNE-treated serum-starved ARPE-19 cells. The JNK inhibitor SP600125 (SP), the MEK1/2 inhibitor PD98059 (PD) and the p38 MAPK inhibitor SB203580 (SB) had no protective effect on the cell viability ((**a)**: MTT assay and (**b**) LDH assay) of serum-starved ARPE-19 cells stimulated with HNE. Release of the cytokine IL-6 (**c**) was decreased only by PD, whereas IL-8 (**d**) levels were reduced by both PD and SP but increased by SB when compared to cells exposed to HNE and the solvent DMSO. Cell viability was normalized to untreated controls while the cytokine levels were compared to HNE + DMSO-exposed positive controls. Addition of HNE + DMSO reduced the release of IL-6 and IL-8 compared to untreated control (Suppl. Fig. 4). Results are shown as scatterplots with median and are combined from 3 independent experiments with 3–4 parallel determinations per group/experiment. *denotes p < 0.05, ***denotes p < 0.001, ns denotes not statistically significant, Mann–Whitney *U*-test.

In order to test the role of the inhibition of MAPK on the release of IL-6 and IL-8 from HNE-treated ARPE-19 cells, we inhibited MAP kinases JNK and p38, as well as MAP kinase kinase MEK1/2 by adding specific MAPK inhibitors 1 hour after the HNE treatment. Inhibition of either of these MAPKs conferred no protective effect on the cellular viability of HNE-treated ARPE-19 cells (Fig. 6a,b). Instead, the inhibition of p38 inhibition seemed to decrease the metabolic activity and compromise the cellular membrane integrity, which was determined using the MTT assay and the LDH assay, respectively (Fig. 6a,b). The inhibition of p38 also resulted in increased IL-8 levels appearing in the medium (Fig. 6d). On the other hand, the inhibition of ERK1/2 by blocking its upstream regulator MEK1/2 decreased the release of both IL-6 and IL-8 (Fig. 6c,d). Furthermore, the inhibition of JNK decreased the levels of IL-8 but exerted no effect on the IL-6 production (Fig. 6c,d). Our present results suggest that JNK and ERK1/2 play important roles in the anti-inflammatory effects of fisetin and luteolin.

### Anti-inflammatory properties of fisetin and luteolin are not mediated by SIRT1 in HNE-exposed ARPE-19 cells

**Figure 7:**
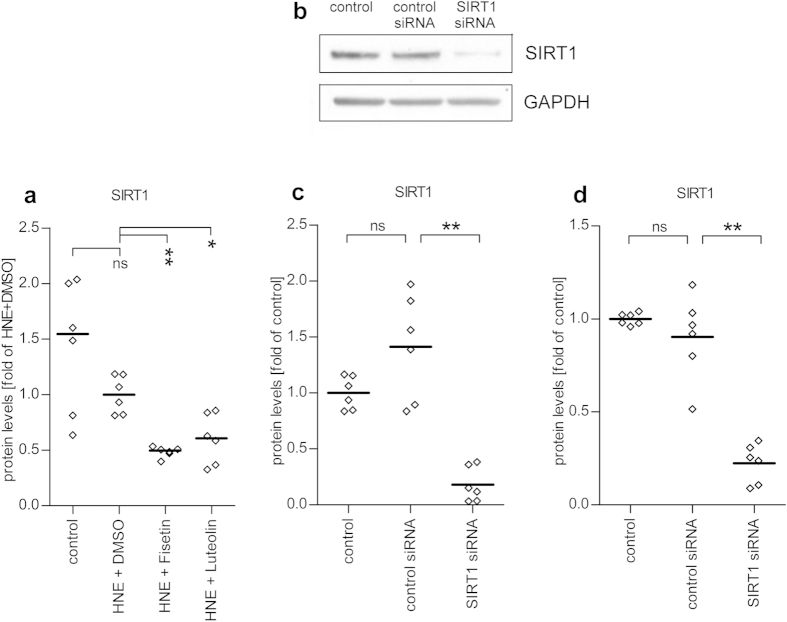
Effects of fisetin and luteolin on SIRT1 levels in HNE-treated serum-starved ARPE-19 cells and efficacy of SIRT1 siRNA in ARPE-19 cells. Both fisetin and luteolin decreased intracellular SIRT1 levels when compared to untreated controls or cells treated with HNE and the solvent DMSO alone (**a**). Results were obtained using SIRT1 specific ELISA. Also SIRT1 siRNA effectively decreased protein levels in ARPE-19 as measured by both Western Blot (**b,c**) or ELISA methods (**d**). Bands are from one representative Western blot (whole blots in Suppl. Fig. 5) and quantitative analysis represents a combination from 3 independent experiments with 2 parallel determinations per group/experiment. Data in graphs is shown as scatterplots with median. *denotes p < 0.05, **denotes p < 0.01, ns denotes not statistically significant, Mann–Whitney *U*-test.

**Figure 8:**
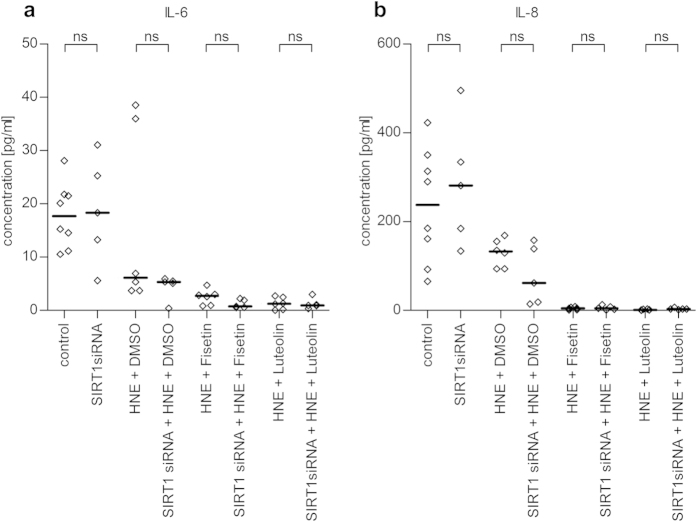
Effect of SIRT1 knock-out on the anti-inflammatory properties of fisetin and luteolin. There were no differences in IL-6 (**a**) and IL-8 (**b**) levels between SIRT1 knock-down samples and those still expressing SIRT1. Results are shown as a scatterplot with median and represent a combination from 3 independent experiments with 2–4 parallel determinations per group/experiment. *denotes p < 0.05, **denotes p < 0.01, ns denotes not statistically significant, Mann–Whitney *U*-test.

Fisetin and luteolin have been associated with the activation of SIRT1, the human analogue of the yeast longevity gene *Sir2* that, in addition to being involved in longevity, has also putative effects on the oxidative stress response and inflammatory signaling^42^,^43^. To establish whether SIRT1 would be involved in the anti-inflammatory effects of fisetin and luteolin, we measured intracellular SIRT1 levels from cell lysates. Surprisingly, we detected a decline in SIRT1 levels in HNE-treated ARPE-19 cells 24 h after co-treatment with either fisetin or luteolin (Fig. 7a). Treatment with HNE and the solvent DMSO alone did not significantly decrease SIRT1 levels (p = 0.3939). These results suggest that down-regulation of SIRT1 might be involved in the anti-inflammatory effects of fisetin and luteolin in HNE-treated ARPE-19 cells, despite descriptions that SIRT1 is an anti-inflammatory protein in other cell lines. However, since the activation of SIRT1 is facilitated by allosteric activators, SIRT1 protein levels do not necessarily correlate with its activity^44^,^45^. We therefore determined whether the downregulation we observed was linked to the anti-inflammatory effects of fisetin and luteolin by transfecting ARPE-19 cells either with specific SIRT1 siRNA or a noncoding control siRNA. Western blotting and ELISA measurements confirmed a significant decrease in the SIRT1 protein levels in ARPE-19 cells 48 h after the transfection (Fig. 7b–d, p = 0.0022 in both ELISA and western blot analysis). Subsequently, siRNA-transfected cells were treated with HNE 48 h post-transfection and then, 1 h later exposed to either fisetin or luteolin, or their solvent DMSO. After 24 h of the polyphenol exposure, the levels of secreted IL-6 and IL-8 were analyzed and no differences were found between the control and SIRT1 siRNA-transfected groups (Fig. 8). This suggests that SIRT1 downregulation is not involved in mediating the anti-inflammatory activities exerted by fisetin and luteolin in HNE-treated ARPE-19 cells.

## Discussion

Under normal conditions, the RPE, a non-dividing cell layer, is unusually resistant to apoptosis and cellular death, even though it is constantly exposed to a high oxygen tension and detrimental phototoxic effects. However, this protection is lost with advanced age and the onset of AMD^46^. Here, we demonstrate that ARPE-19 cells, also known for their high resilience to apoptosis, are more susceptible to the oxidative stress induced by the lipid peroxidation end-product, HNE, when they have been cultured entirely in serum-free medium. Despite their increased sensitivity to toxic stressors and delayed adhesion to and spreading on the surface of culture wells in the absence of serum, ARPE-19 cells still grew to full confluency and exhibited no signs of any disturbed development. This result is in line with an earlier report by Jun *et al*.^47^. They also reported an infrequent cell phenotype with spindle-like extrusions when cells were cultured without serum for 24 hours. We observed this same phenotype, but it was both rare and transient and was also found in cells cultured in serum-containing medium, albeit at earlier time-points.

HNE is an end-product of lipid peroxidation, a process that is induced by oxidative stress. In addition to playing a role as a signaling molecule, HNE induces apoptosis and evokes cellular death if its intracellular levels become too high^17^,^48^. In our study, HNE triggered considerable cell death in stressed ARPE-19 cells as observed by the increased LDH levels in the medium, and decreased metabolic activity measured by the MTT test. We have previously shown that an exposure of ARPE-19 cells to HNE decreases the release of inflammatory cytokines IL-6 and IL-8 to the medium, an effect that is not exclusively due to the high toxicity of HNE^49^. We have speculated that this might result from the ability of HNE to specifically inhibit the ability of the NF-κB subunit p65 to bind to nuclear DNA^39^,^49^. This hypothesis is also in line with the findings of Page *et al*. who reported that HNE could inhibit the NF-κB activation^50^.

Despite the reductions in IL-6 and IL-8 release caused by the HNE exposure alone, addition of fisetin or luteolin effectively further decreased the levels of these pro-inflammatory cytokines. At the same time, fisetin and luteolin protected stressed ARPE-19 cells from HNE-induced cytotoxicity. Additional experiments using bacterial lipopolysaccharide (LPS) as an alternative pro-inflammatory stimulant confirmed the anti-inflammatory effect of fisetin and luteolin (Suppl. Fig. 6). The level of reduction of IL-6 and IL-8 release after fisetin or luteolin treatment was comparable between HNE- and LPS-treated ARPE-19 cells.

In a therapeutic sense, it is interesting that fisetin and luteolin induced advantageous effects even when added after the oxidative insult and that they do so in both confluent monolayers, and fully differentiated ARPE-19 cells (Suppl. Fig. 3). Previous *in vitro* studies have usually used only pretreatment or concurrent treatment of fisetin and luteolin with an oxidative stressor. Hanneken *et al*.^51^ described the ability of both fisetin and luteolin to prevent the oxidative-stress induced cell death if added up to 4 hours after the initiation of oxidative stress. However, these researchers used either H_2_O_2_, a direct oxidant or tert-butyl hydroperoxide (t-BOOH), an inducer of lipid peroxidation, to evoke oxidative stress. Since RPE cells have certain distinctive characteristics i.e. phagocytosis of unsaturated lipids-containing POS, very high metabolic activity, and extensive use of oxygen leading to naturally high levels of HNE^15^,^17^, we considered that HNE would represent a more physiological model of increased oxidative stress.

Unlike previous studies performed with LPS-stimulated macrophages^27^,^52^, we did not observe any effect of fisetin and luteolin on NF-κB activation. This might be attributable to the fact that the chosen oxidative stressor, HNE, decreased p65 binding already by itself, an effect that was not amplified by either fisetin or luteolin. Instead, we found that HNE enhanced the phosphorylation, and therefore the activity of MAPKs p38 and JNK, and the transcription factor CREB, all of which are known to regulate the transcription of IL-6 and IL-8^4^,^5^,^31^,^33^,^37^. Both fisetin and luteolin markedly reduced the phosphorylation levels of these proteins, as well as the phosphorylation of ERK1/2 when compared to HNE + DMSO-treated cells, suggesting that these proteins could contribute to the additional reduction in IL-6 and IL-8 levels observed after the fisetin and luteolin treatments. These results are in line with several previous reports that have described the effects of either fisetin or luteolin on the MAPK activity in a wide range of different cell lines and using different activators of oxidative stress or inflammation^3^,^12^,^18^,^19^,^20^,^21^,^22^,^24^,^26^,^27^,^28^,^29^,^31^,^32^,^34^,^35^,^36^,^37^. It should be noted that the results have depended greatly on which cell line was tested and which stressor used. As far as we are aware, this is the first report of the effects of fisetin and luteolin on the activity of MAPKs in ARPE-19 cells.

In order to better evaluate the importance of the separate MAPKs in the inflammatory response of stressed ARPE-19 cells, we measured the effects of specific MAPK inhibitors on the release of two pro-inflammatory cytokines, IL-6 and IL-8. We found that inhibition of p38 MAPK exerted no positive effect on the release of IL-6 or IL-8, which is in contrast to findings by Wang *et al*.^53^. Only JNK or ERK1/2 inhibitors were able to decrease the release of IL-8 in ARPE-19 cells. The differences between previous results and the present values might be due to the stressed and serum-starved state of ARPE-19 cells in this study. It is also worth noting that Kang *et al*.^31^ reported luteolin to have a greater effect on the inhibition of JNK and ERK1/2 than their specific inhibitors. This might partly explain the lack of efficacy of the specific inhibitors used in our experiments.

Fisetin and luteolin are known to be SIRT1 activators^54^,^55^ and therefore, we studied whether this property might have consequences for inflammatory signaling in stressed ARPE-19 cells. SIRT1 is a well-characterized protein, known to exert numerous influences on metabolism and aging^42^,^56^. SIRT1 activation has been shown to positively affect the development of age-related diseases like Alzheimer’s disease in a mouse model^45^ and it has also been demonstrated to decrease the cataract formation during normal aging in mice^45^. Resveratrol, a well-known SIRT1 activator was able to decrease inflammation in endotoxin induced uveitis in mice by upregulating SIRT1 activity in the RPE^57^. It also reduced the volume of choroidal neovascularization and decreased inflammation in the eyes of mice undergoing laser-photocoagulation in a manner that was dependent on the activation of AMPK, a downstream target of SIRT1^58^. At the molecular level, SIRT1 is known to control inflammation both directly and indirectly by regulating the NF-κB-related signaling^43^,^59^.

To our surprise, we found that the addition of fisetin or luteolin to HNE-treated ARPE-19 cells resulted in a reduced expression of SIRT1. However, fisetin and luteolin, along with most other known SIRT1 activators are allosteric activators of SIRT1, and can achieve an increase in SIRT1 activity without increasing the levels of SIRT1 protein in cells^44^,^55^,^60^. In our studies to determine whether the decrease in SIRT1 levels had an effect on the inflammatory reactions in ARPE-19 cells, we found that the knock-down of SIRT1 by specific siRNAs had no effect on the inflammatory response or on fisetin’s and luteolin’s abilities to reduce the level of inflammation. These results are in line with the findings that fisetin and luteolin did not affect the DNA-binding activity of the NF-κB subunit p65 and indicate that the anti-inflammatory actions of the polyphenols in ARPE-19 cells are independent of SIRT1.

**Figure 9:**
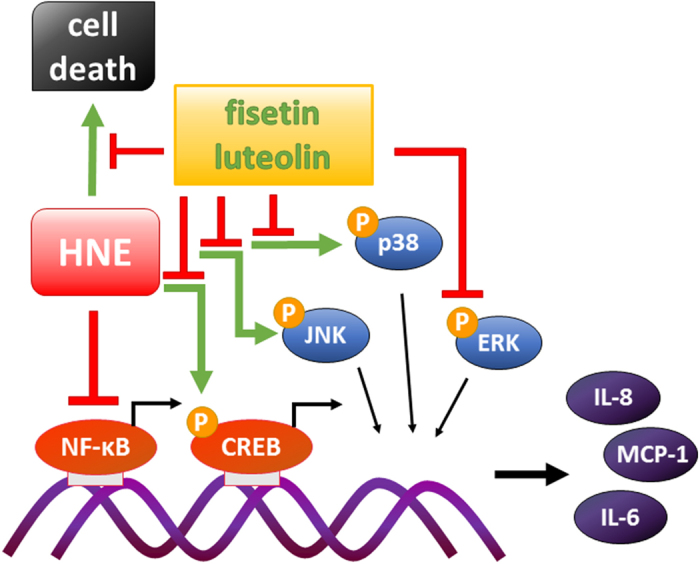
Summary of the effects of fisetin and luteolin on serum-starved and HNE-treated ARPE-19 cells. Addition of HNE to serum-starved cells was strongly cytotoxic, and caused decreased release of pro-inflammatory cytokines IL-6 and IL-8 probably trough the inhibition of NF-κB signaling, as discussed previously. Fisetin and luteolin protected the cells from HNE-induced cytotoxicity and induced a strong additional reduction in the IL-6, IL-8, and MCP-1 levels even when added after the HNE exposure. Analysis of signaling protein amounts and phosphorylation levels together with inhibition studies suggest that fisetin and luteolin suppress the release of pro-inflammatory cytokines by decreasing the activation of CREB, JNK, and ERK1/2.

Overall, fisetin and luteolin show a promising ability to protect RPE cells from oxidative stress-induced cell death and to potently downregulate inflammatory reactions in these cells by decreasing the activity of the transcription factor CREB and the MAPKs p38 MAPK, JNK, and ERK1/2 (Fig. 9). Nonetheless, neither NF-κB nor SIRT1 was involved in the inflammatory regulation. These results offer us valuable insights into the signaling underlying RPE-derived inflammatory reactions and thereby into the pathogenesis of AMD.

## Additional Information

**How to cite this article**: Hytti, M. *et al.* Fisetin and luteolin protect human retinal pigment epithelial cells from oxidative stress-induced cell death and regulate inflammation. *Sci. Rep.* **5**, 17645; doi: 10.1038/srep17645 (2015).

## Supplementary Material

Supplementary Figures

Supplementary Video 1

Supplementary Video 2
