# Supplementary material for: Fisetin and luteolin protect human retinal pigment epithelial cells from oxidative stress-induced cell death and regulate inflammation
Source: Sci Rep. 2015 Dec 1;5:17645. doi: 10.1038/srep17645 (PMC4664957; doi:10.1038/srep17645)
Supplement: Supplementary Figures [file srep17645-s1.doc]

**Fisetin and luteolin protect human retinal pigment epithelial cells from oxidative stress-induced cell death and regulate inflammation**

**Maria Hytti^1, 2^, Niina Piippo^1, 2^, Eveliina Korhonen^1, 2^, Paavo Honkakoski^1^, Kai Kaarniranta^2, 3^ and Anu Kauppinen^1, 3, *^**

^1^School of Pharmacy, Faculty of Health Science, University of Eastern Finland, P.O.B. 1627, FI-70211, Kuopio, Finland

^2^Department of Ophthalmology, Institute of Clinical Medicine, University of Eastern Finland, P.O.B. 1627, FI-70211 Kuopio, Finland

^3^Department of Ophthalmology, Kuopio University Hospital, P.O.B. 100, FI-70029 KYS, Finland

*anu.kauppinen@uef.fi


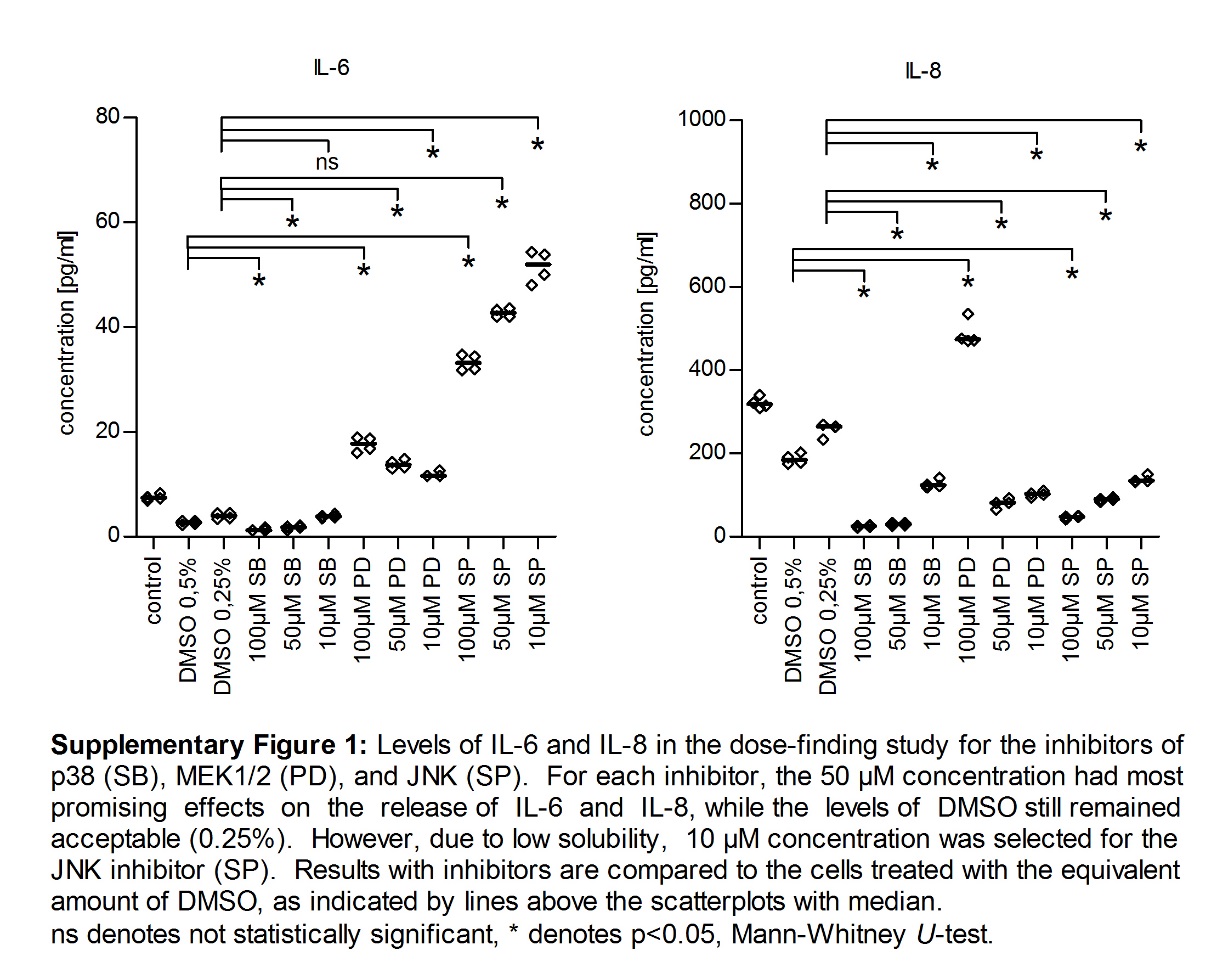


**Supplementary figure 1:** Levels of IL-6 and IL-8 in the dose-finding study for the inhibitors of p38 (SB), MEK1/2 (PD) and JNK (SP). For each inhibitor the 50µM concentration had most promising effects on the release of IL-6 and IL-8 with acceptable levels of DMSO (0.25%). However, due to low solubility, a 10µM concentration was selected for the JNK inhibitor (SP). Results are compared to groups with equimolar DMSO concentrations and are represented as scatterplots with median. ns denotes not statistically significant, * denotes p<0.05, Mann-Whitney *U*–test.


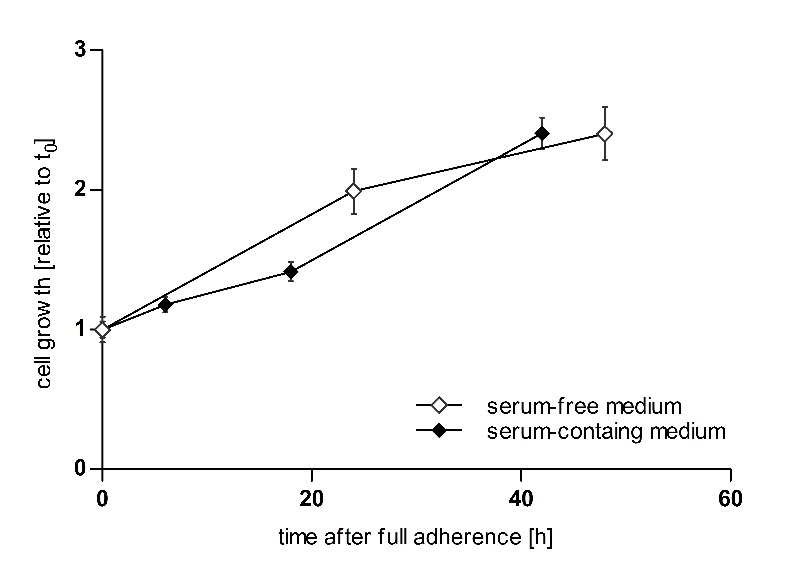


**Supplementary figure 2:** Influence of serum on the growth rate of ARPE-19 cells. After full adhesion, the growth curves in both medium types were very similar. Data is shown relative to the cell count at the time-point of full adhesion of ARPE-19 cells to the plate (6 h for cells in serum-containing medium and 24 h for cells in the serum-free medium). Results are presented as mean ± SEM of 3-5 independent experiments with 4 replicates per time-point/group.

**
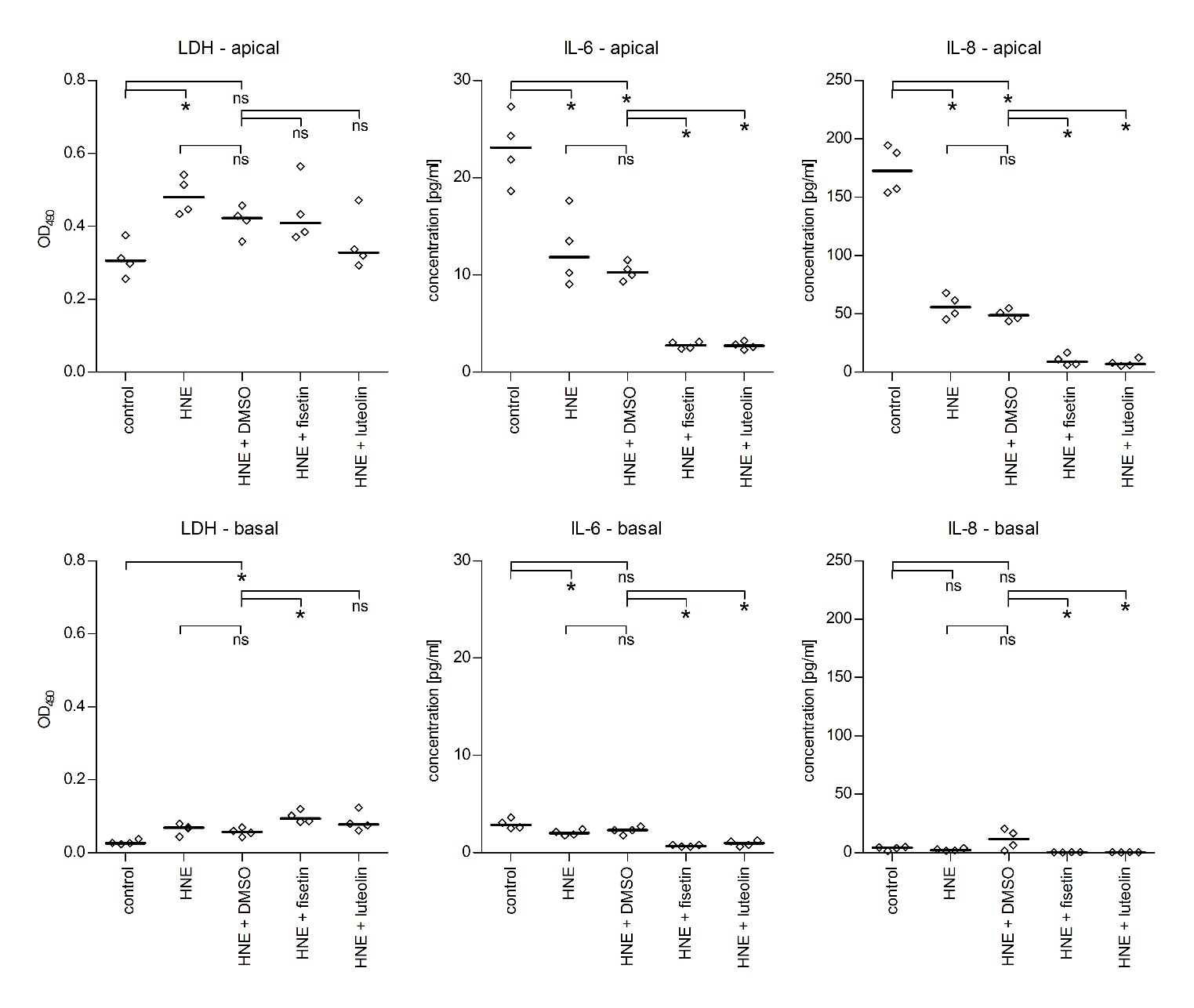
**

**Supplementary Figure 3:** Effects of fisetin and luteolin on the release of LDH, IL-6 and IL-8 from differentiated ARPE-19 cells exposed to HNE. Similarly to the results observed in confluent, but not fully differentiated, monolayers, HNE treatment decreased the release of IL-6 and IL-8 to both the basal and the apical side of RPE cells. Fisetin and luteolin further reduced the release of both inflammatory cytokines. HNE also led to an increased release of LDH to the medium but unlike in confluent monolayers fisetin and luteolin were unable to reduce this release. Results are shown as scatterplots with median from 4 parallel samples per group. * denotes p < 0.05 and “ns” denotes not statistically significant, Mann-Whitney *U*–test.


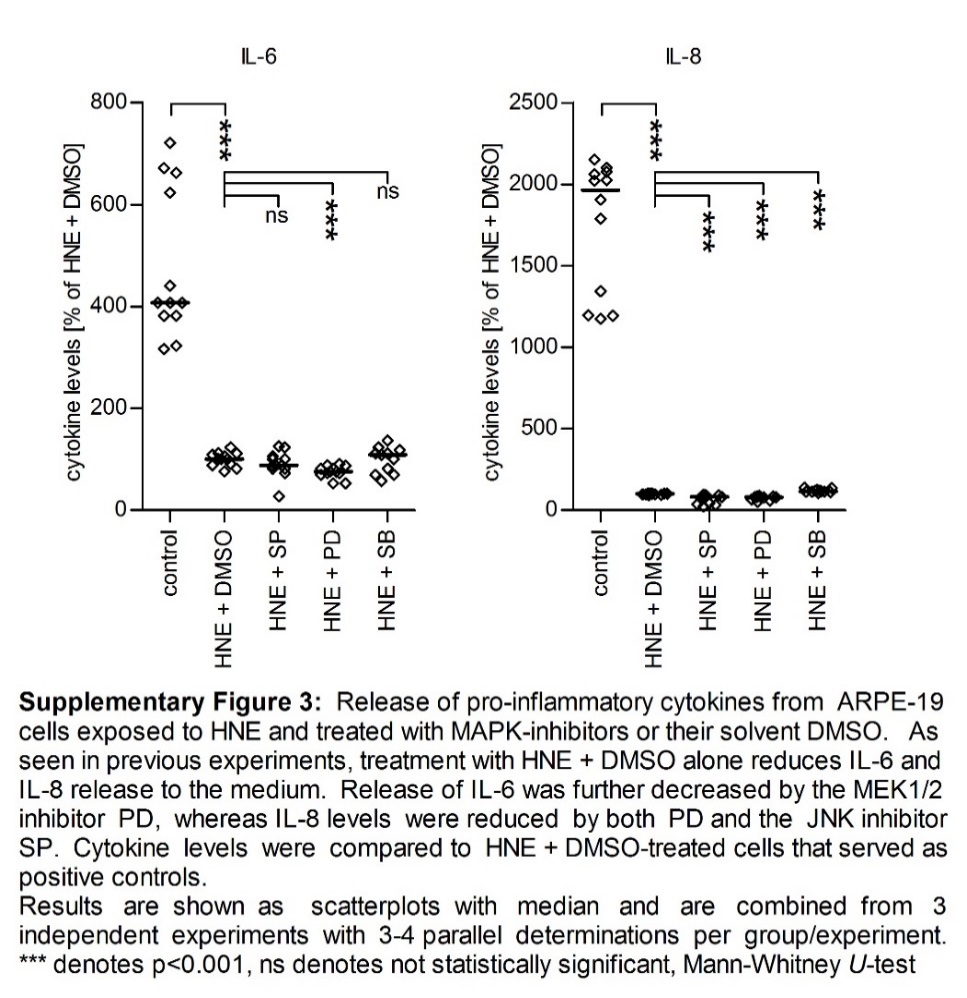


**Supplementary Figure 4:** Release of pro-inflammatory cytokines from ARPE-19 cells exposed to HNE and treated with MAPK-inhibitors or their solvent DMSO. As seen in previous experiments, treatment with HNE + DMSO alone reduces IL-6 and IL-8 release to the medium. Release of IL-6 was further decreased by the MEK1/2 inhibitor PD, whereas IL-8 levels were reduced by both PD and the JNK inhibitor SP. Cytokine levels were compared to HNE + DMSO-treated cells that served as positive controls. Results are shown as scatterplots with median and are combined from 3 independent experiments with 3-4 parallels per group/experiment. ns denotes not statistically significant, *** denotes p<0.001, Mann-Whitney *U*–test

SIRT1 GAPDH


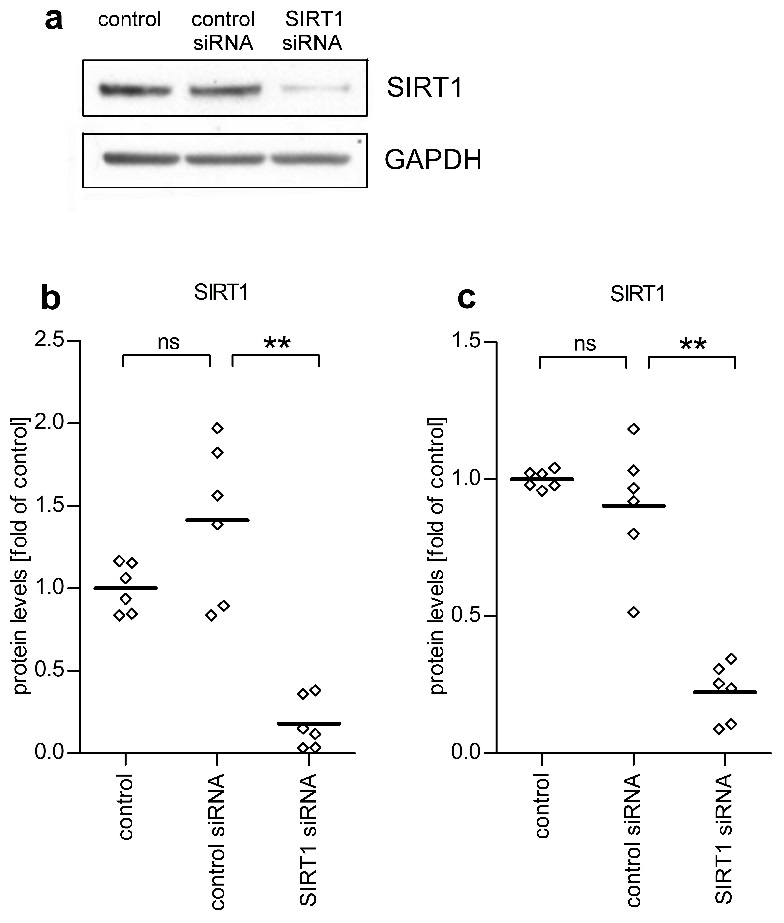

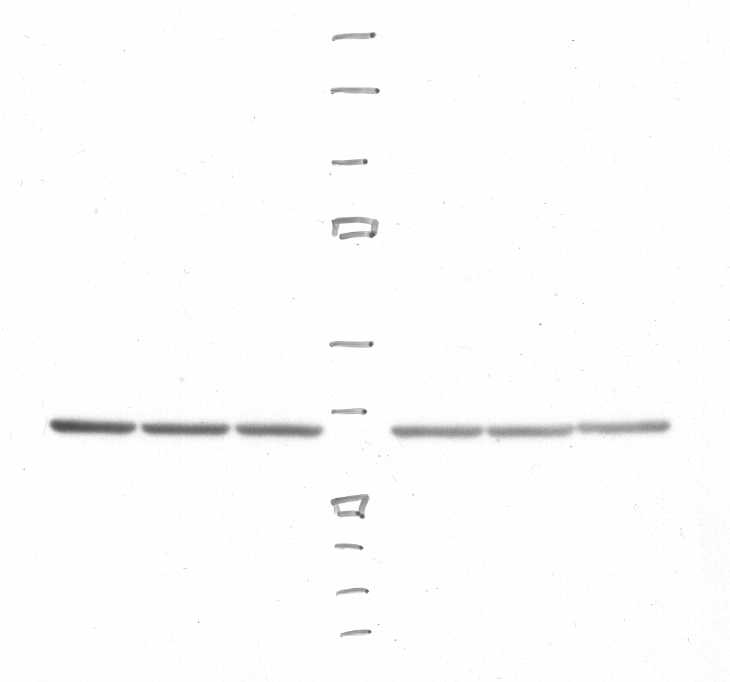

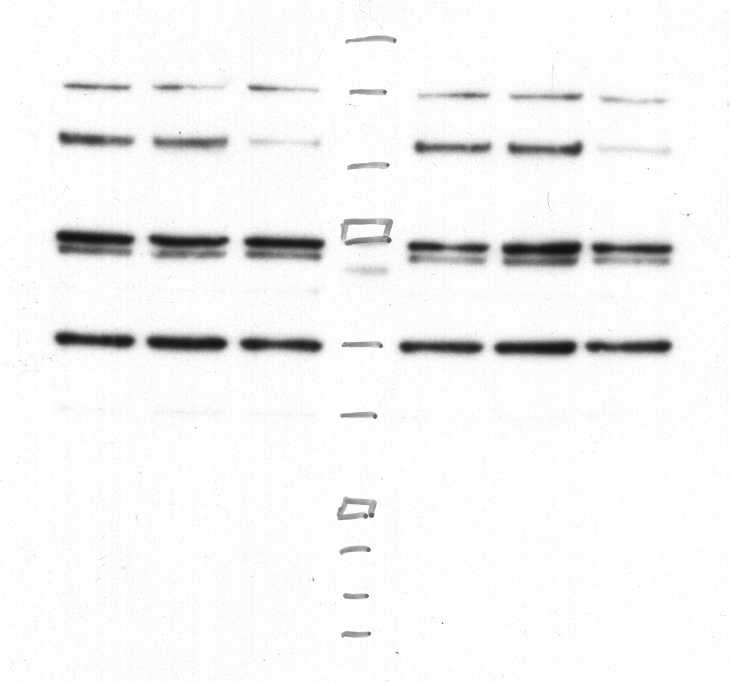

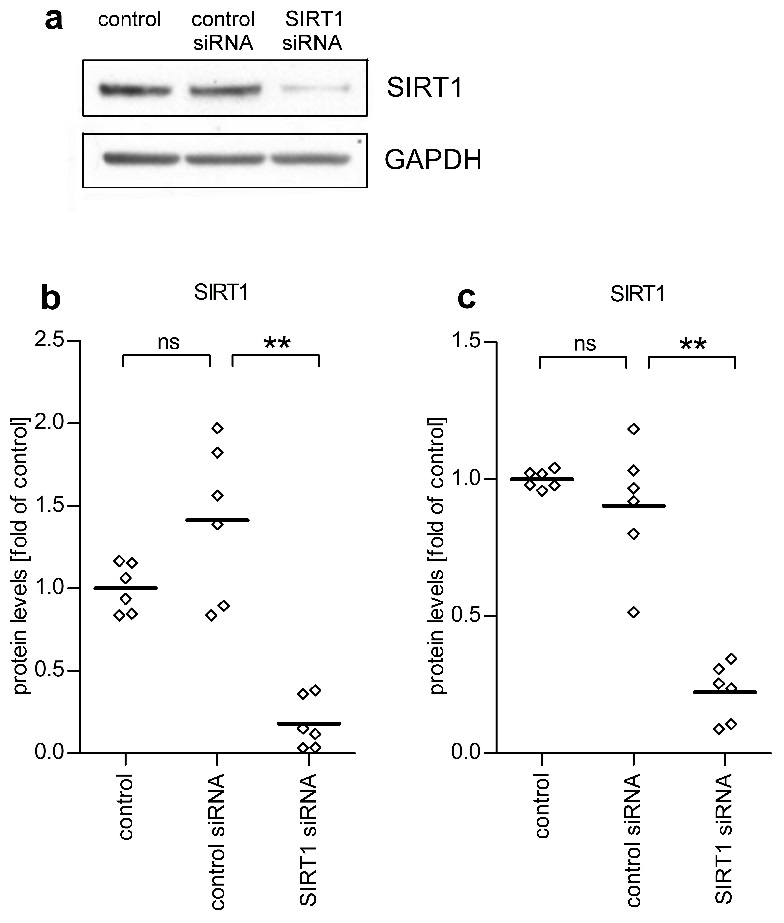

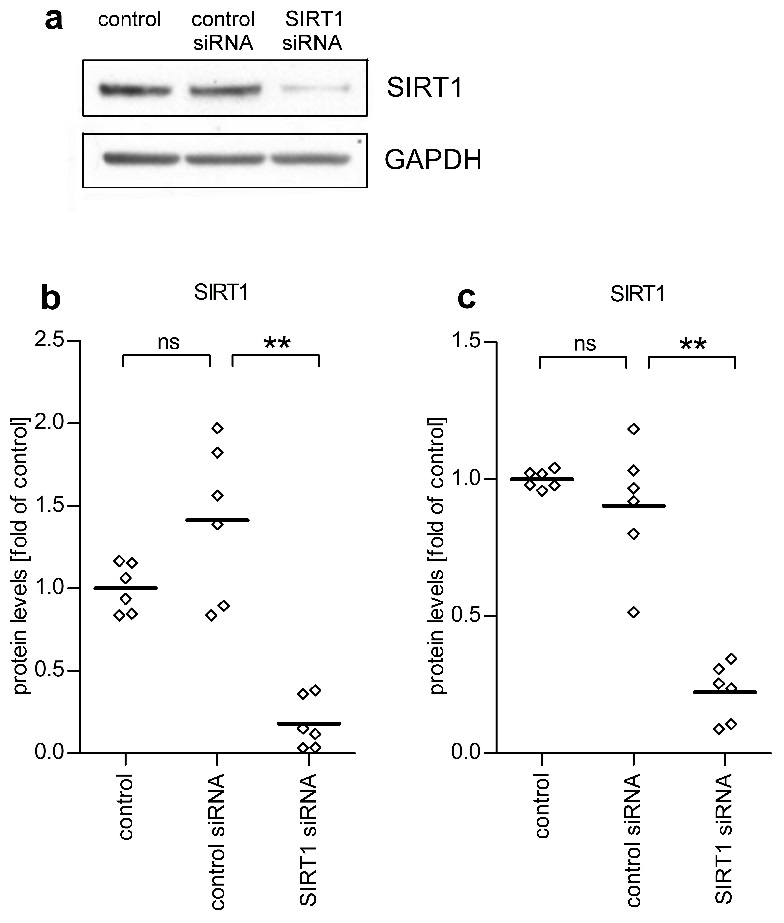

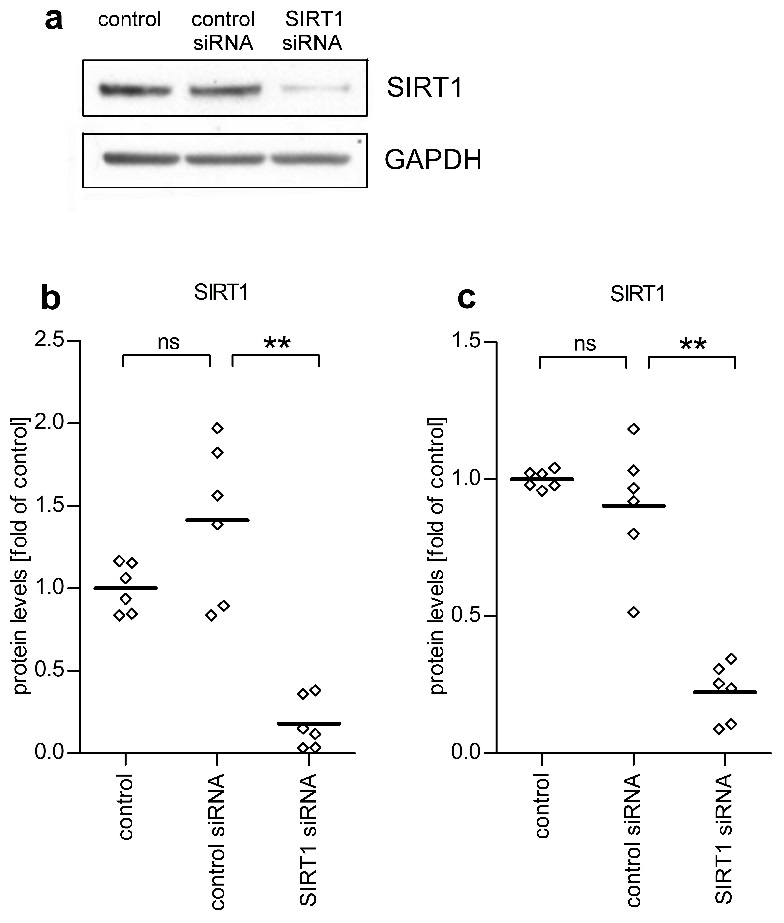


250 kDa

150 kDa

100 kDa

75 kDa

50 kDa

37 kDa

25 kDa

20 kDa

15 kDa

10 kDa

**Supplementary Figure 5:** Western blot analysis of the efficacy of SIRT1 siRNA. SIRT1 siRNA can efficiently downregulate the expression of SIRT1 (left) without effect on the levels of the internal control GAPDH (right).

Grey outline signifies size of the membrane, areas surrounded by black dotted line are cropped images included in Figure 9 of the article. Expected size of SIRT1 = 110kDa, expected size of GAPDH = 30-40 kDa. Marker sizes indicated in grey between blots.

**Supplementary Figure 6:** Effect of fisetin and luteolin treatment on serum-starved, LPS-treated ARPE-19 cells. LPS-stimulation led to a strong increase in both IL-6 and IL-8 levels, which was not influenced by presence or absence of the solvent DMSO. Fisetin and luteolin both decreased the release of IL-6 and IL-8 to levels below the values of unstimulated controls. Fisetin and luteolin, but not LPS caused an increase in the release of LDH from the cells into the medium. Results are combined from 3 independent experiments with 4 parallels per group per experiment and shown as scatterplots with median. ns denotes not statistically significant, * denotes p< 0.05, *** denotes p< 0.001, Mann-Whitney *U*–test.

**Legends for Supplementary Videos**

**Supplementary Video 1:** Time-lapse video of cells cultured in serum-containing medium between 2.5 and 13 hours after splitting. Cells are quick to attach to the surface, especially in the vicinity of other cells and spread out. Images were taken every ten minutes for 10.5 hours and combined to create an animated representation. Time elapsed in hours is presented in the upper right corner.

**Supplementary Video 2:** Time-lapse video of cells cultured in serum-free medium between 2.5 and 13 hours after splitting. Cells take longer to attach to the surface and retain their rounded phenotype for longer, spreading out slowly once in the vicinity of other cells. Images were taken every ten minutes for 10.5 hours and combined to create an animated representation. Time elapsed in hours is presented in the upper right corner.
